# Encountering epidemic effects of leaf spot disease (*Alternaria brassicae*) on *Aloe vera* by fungal biocontrol agents in agrifields—An ecofriendly approach

**DOI:** 10.1371/journal.pone.0193720

**Published:** 2018-03-26

**Authors:** Swapan Kumar Ghosh, Subhankar Banerjee, Sujoy Pal, Niloy Chakraborty

**Affiliations:** Post-Graduate Dept. of Botany, Ramakrishna Mission Vivekananda Centenary College, Kolkata, West Bengal, India; Karnatak University, INDIA

## Abstract

*Aloe vera* (L.) Burm.f. is a highly important and extensively cultivated medicinal plant and that is also extensively used in the cosmetic industry. It has been frequently reported to suffer from *Alternaria* leaf spot disease in various parts of the world. Various fungicides used to combat this disease, have deleterious effects on the environment and on pharmacologically important constituents of *Aloe vera*. To avoid the harmful effects of fungicides an ecofriendly approach has been adopted here. A weekly survey was conducted during 2013–2015 in and around North 24 Parganas (West Bengal) to obtain the percentage of disease index (PDI). For biological control of the disease, screening of the antagonistic efficacy of biocontrol agents was carried out through the *in vitro* dual-culture-plate method and scanning electron microscopy (SEM) was used to study the mechanism. The *in vitro* effects of fungicides on the radial growth of the pathogen were evaluated through the poison food method and were compared with potent antagonistic fungi. Field application of potent antagonistic fungi was conducted through the dip-and-spray method. The results showed that, the PDI peaked during the hot and humid conditions of May to September (76.57%–98.57%) but decreased during the winter, December-January (35.71–46.66%). *Trichoderma asperellum* exerted the greatest inhibition of the radial growth of *A*. *brassicae* acting through non volatile (70.39%) and volatile metabolites (72.17%). A SEM study confirmed the hyperparasitic nature of *T*. *asperellum* through hyphal coiling-*T*. *asperellum* was similar to 2% blitox-50 (73.92%) and better than 2% bavistin (59.77%) (*in vitro*). In agricultural field trials (2013–15), *Trichoderma* application restricted the disease to the smallest area (PDI 24.00–29.33%) in comparison to untreated plots (73.33%). In conclusion, saplings treated with the dip method (10^8^ spores / mL) and sprayed 4 times with a spore suspension of biocontrol agents such as *T*. *asperellum*, *T*. *viride* and *T*. *harzianum*, standardized at a rate of 2.5 L / plot (36 sq ft) (10^8^ spores/ mL) are suggested for the ecofriendly management of this epidemic leaf spot disease of *Aloe vera* in agricultural fields.

## Introduction

*Aloe vera* (L.) Burm.f. (*Aloe barbadensis*) belongs to the family Aloeaceae and is an extensively cultivated medicinal plant worldwide, ranging from tropical to temperate regions. Many herbal drugs and drinks have been formulated from *A*. *vera* for the maintenance of good health. In the cosmetic industry. *Aloe* spp. are used in the production of soap, shampoo, hair wash, tooth paste and body creams [1]. Additionally, *A*. *vera* gel has been reported to be very effective for the treatment of sores and wounds, skin cancer, skin disease, colds and coughs, constipation, piles, asthma, ulcer, diabetes and various fungal infections [1,2,3,4]. The plant *Aloe vera* has been frequently reported to have *Alternaria* leaf spot disease, both in India and in various other parts of the world. [5,6,7,8,9]. Many fungal pathogens are responsible for the production of mycotoxins that alter the potentiality of this highly important medicinal plant [10]. Chemical control of these diseases is not an ideal solution to this situation, as chemicals themselves can exert adverse effects on the pharmacologically important and other economically important products of medicinally essential plants. Therefore, biological control is best suited to the scenario. Generally Thiram, Arasan[11], Dithane M-45, Bavistin, Dithane Z-78, Difoltan, Blitox-50 and Bordeaux mixture [12,13] are used to combat *Alternaria* disease. However, the prolonged use of these chemicals is environmentally hazardous and toxic to humans. Non-chemical or eco-friendly methods are now popular in developed countries (such as the USA and -U.K.). Seed priming of tomato with plant growth promoting rhizobacteria (PGPR) such as *Pseudomonas aeruginosa*, *P*. *putida*, *Bacillus subtilis*, *B*.*cereus*, *Azotobacter chroococcum* are reported to increase seed germination, seedling vigor, fruit size, chlorophyll content, IAA production, and rhizospheric phosphate solubilization, which in turn improve plant health and growth. These PGPR strains induce plants’ defences through upregulation of defense-related enzymes such asperoxidise, polyphenol oxidase, glucanase and chitinase along with the production of HCN, siderophore which act as the inducers against *Alternaria*-derived early blight disease in tomato and / or other plant disease resistance involving Systemic Acquired Resistance (SAR) / Induced Systemic Resistance (ISR) mechanisms[14]. Seed treatment with the combination of 3 mg/mL of crude oligosacchharides from the cell wall of *Alternaria solani* and a specific strain of *Bacillus subtilis*, TN_Vel-35, showed significant increases in seed germination and,- seedling vigor, along with enhanced accumulation of defense-related enzymes such aspolyphenol oxidase and peroxidase in comparison to control tomato plants and thus improves plant defense against early blight disease of tomato [15]. A variety of alternative approaches have been adopted among which the use of fungal biocontrol agents such as *Trichoderma* and *Beauveria* has been widely implemented for the management of fungal diseases on crop plants. The application of *Trichoderma* has proven fruitful against many soil borne and foliar pathogens [16,17,18]. Recently formulations from *T*. *viride*, *T*. *harzianum*, and *T*. *virens* have been frequently used as soil applications that, alone or in combination, provided maximum efficacy against diseases of cereals, vegetables, pulses, spices and fruits [19,20,18].

Therefore, the main objectives of this work are the *in vitro* control of the causal organism of *Aloe* leaf spot disease by the antagonistic efficacy of various biocontrol agents such as *Trichoderma viride*, *T*. *harzianum*, *T*. *asperellum*, *T*. *longibrachiatum* and *Beauveria bassiana* and application of potential biocontrol agents to fields, focusing on a lab-to-land approach to combat the devastating leaf spot disease.

## Results and discussion

### Symptoms of the disease

**Fig 1:**
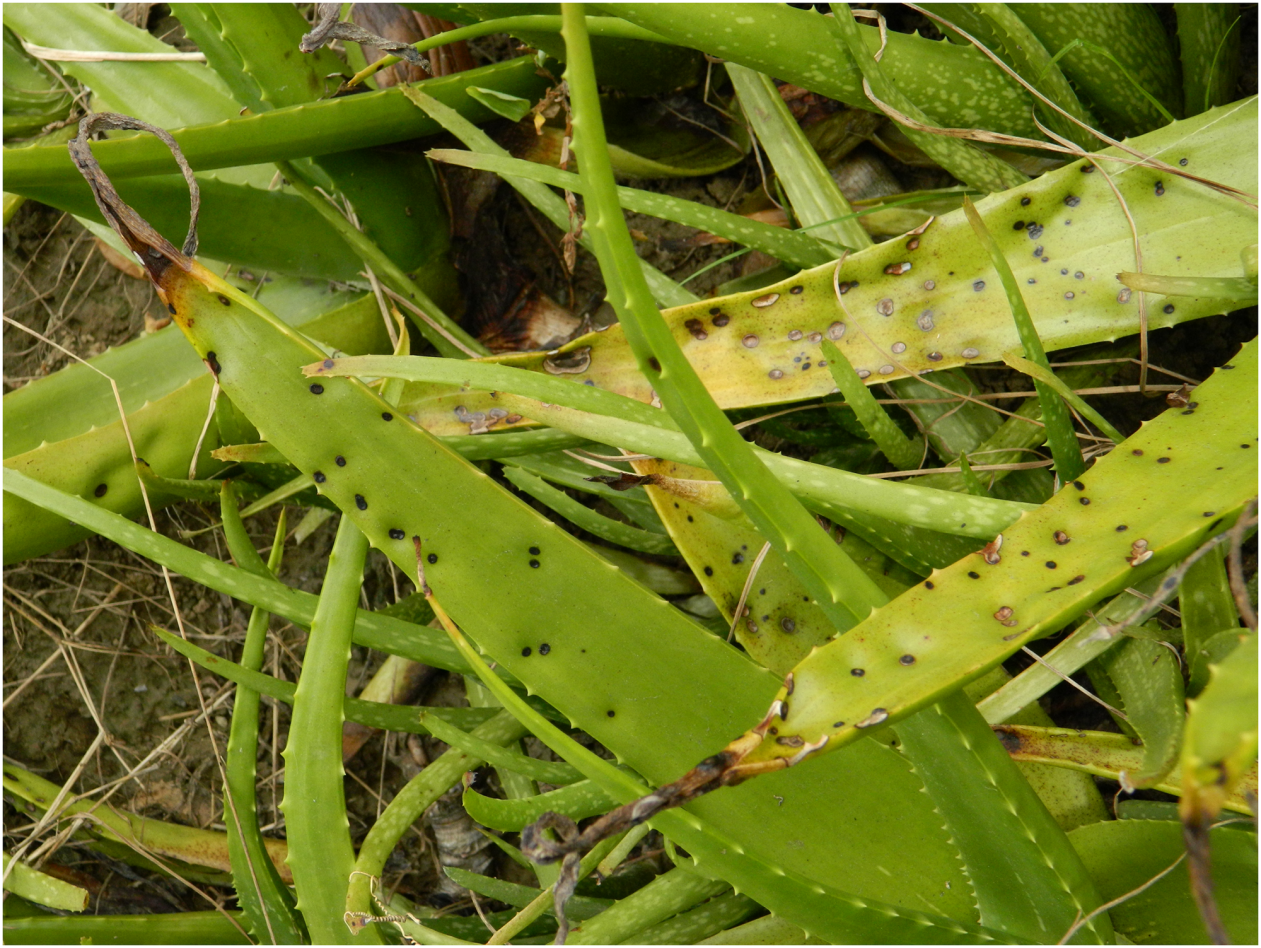
An *Aloe vera* plant showing symptoms of leaf spot.

**Fig 2:**
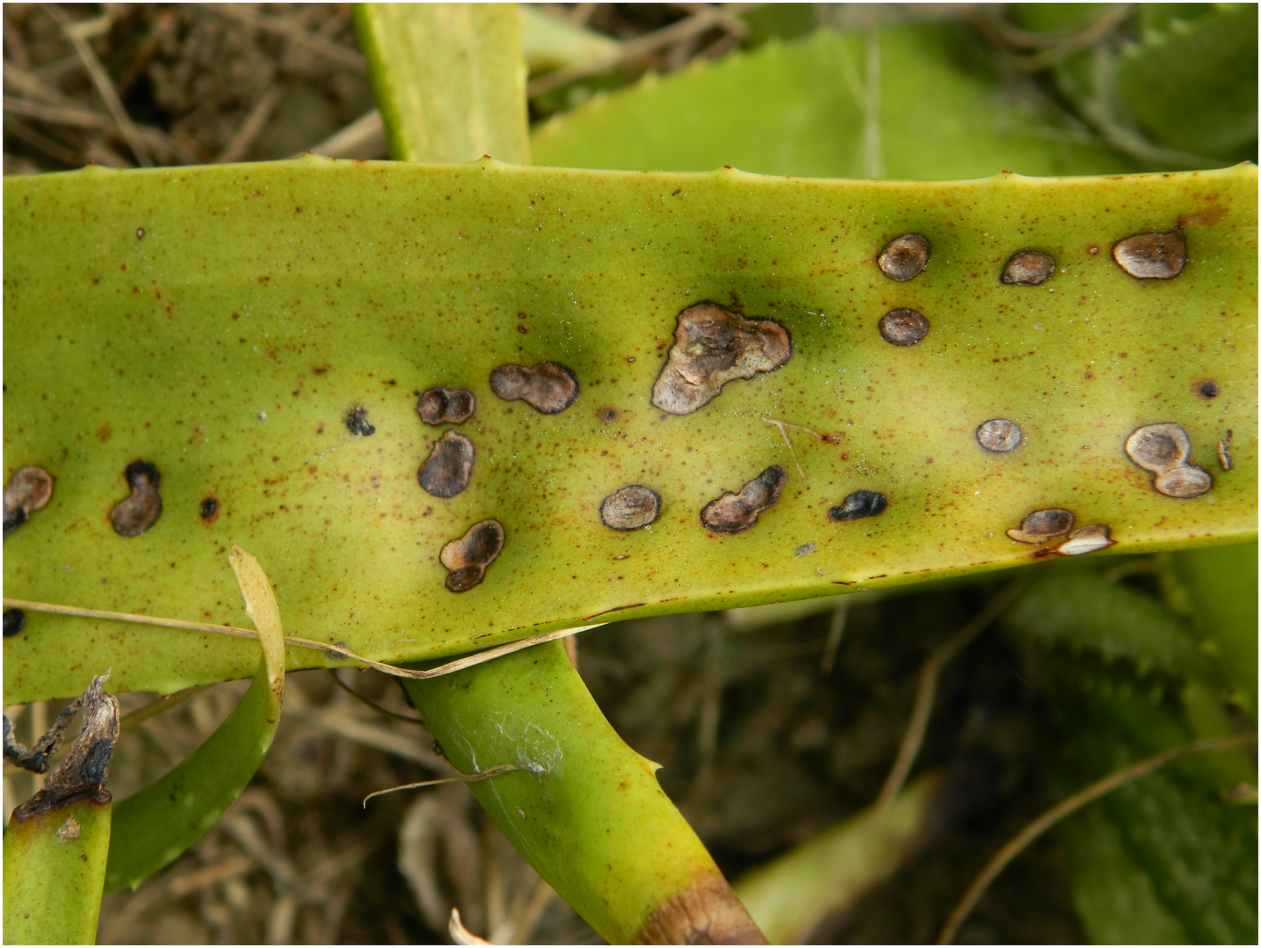
A close-up view of leaf spot symptoms on an *Aloe vera* leaf.

The symptoms appear on the leaves in the form of small dark brown necrotic spots on both sides that gradually become larger, eventually covering an area of 2–8 cm in diameter. The spots gradually coalesce and the infected areas transform from dark brown to black. The leaf surface becomes covered with numerous such lesions, which in turn become rotten and dried within 4-7days, and each lesion finally develops into a central cup shaped depression with a depth of 5–8 mm. (Figs 1 and 2), indicating the presence of leaf spot [5].

On the basis of the microscopic characteristics of pathogen cultures, PCR amplification of ITS1-5.8S-ITS2 from the rDNA of the isolated pathogen, and gene sequencing through the BLAST analysis of a 510-bp sequence, 100% homology with the *Alternaria brassicae* strain HYMS01 (GenBank Accession No. JX857165.1) from NCBI was obtained. The gene sequence was submitted to the NCBI gene bank, was incorporated into GenBank under accession no. KJ022772.1 and was published in NCBI Genbank on March, 23^rd^, 2014 [5].

Through asurvey conducted in and around several regions of North 24 Parganas, specifically, Barasat, Naihati, Basirhat, Mohishbathan, Barrackpore, Nilganj, Haroa, Basanti, Duttapukur, Bongaon, Habra, Kalyani, Halishahar, Taki, Hingalganj, Nahata and Gopalnagar from 2013 to 2015, leaf spot disease of *Aloe vera* was recorded in all the locations under survey at all times of year.

**Fig 3:**
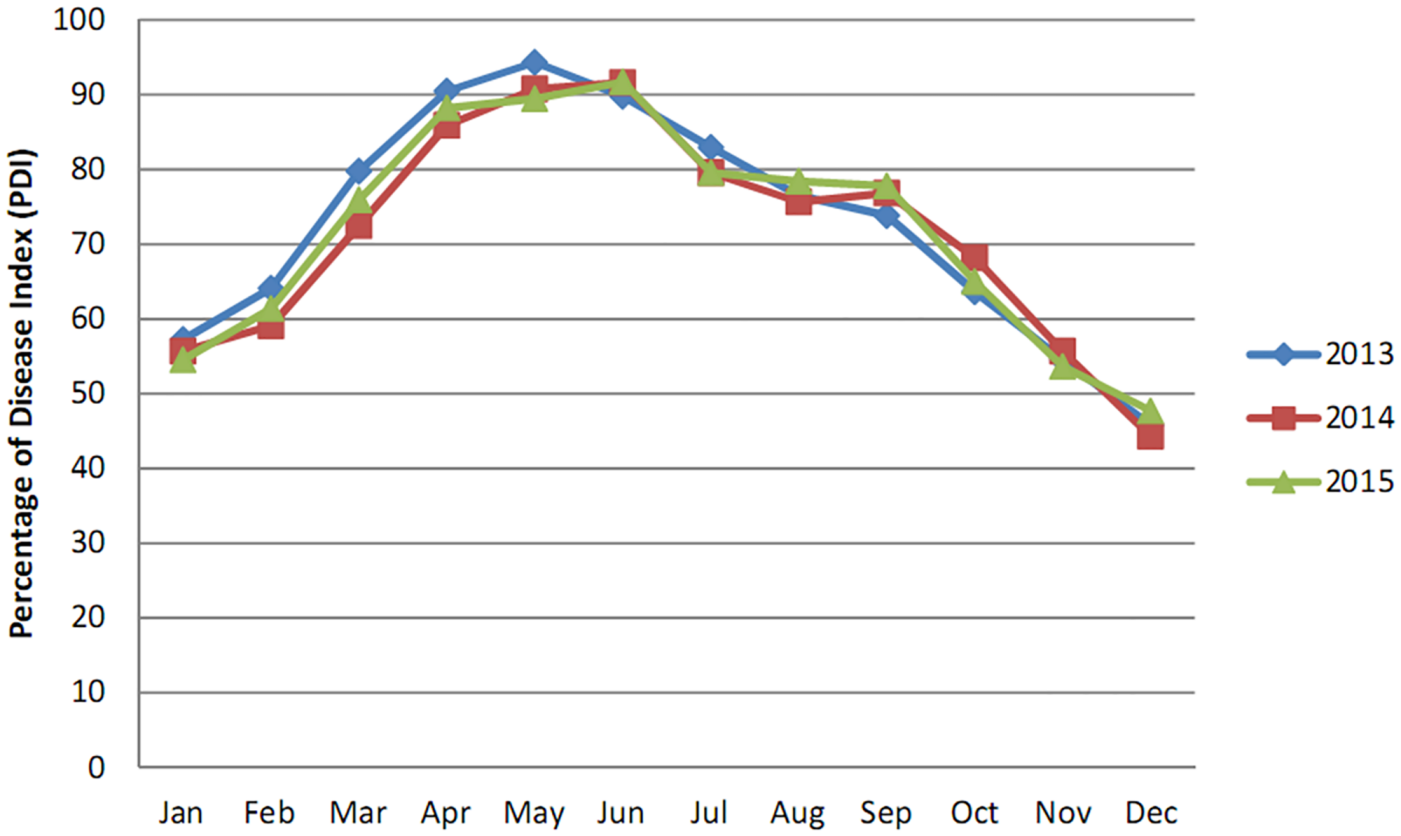
Monthly disease progression curve of *Aloe* leaf spot disease from January, 2013 to December, -2015 in Nilganj.

**Fig 4:**
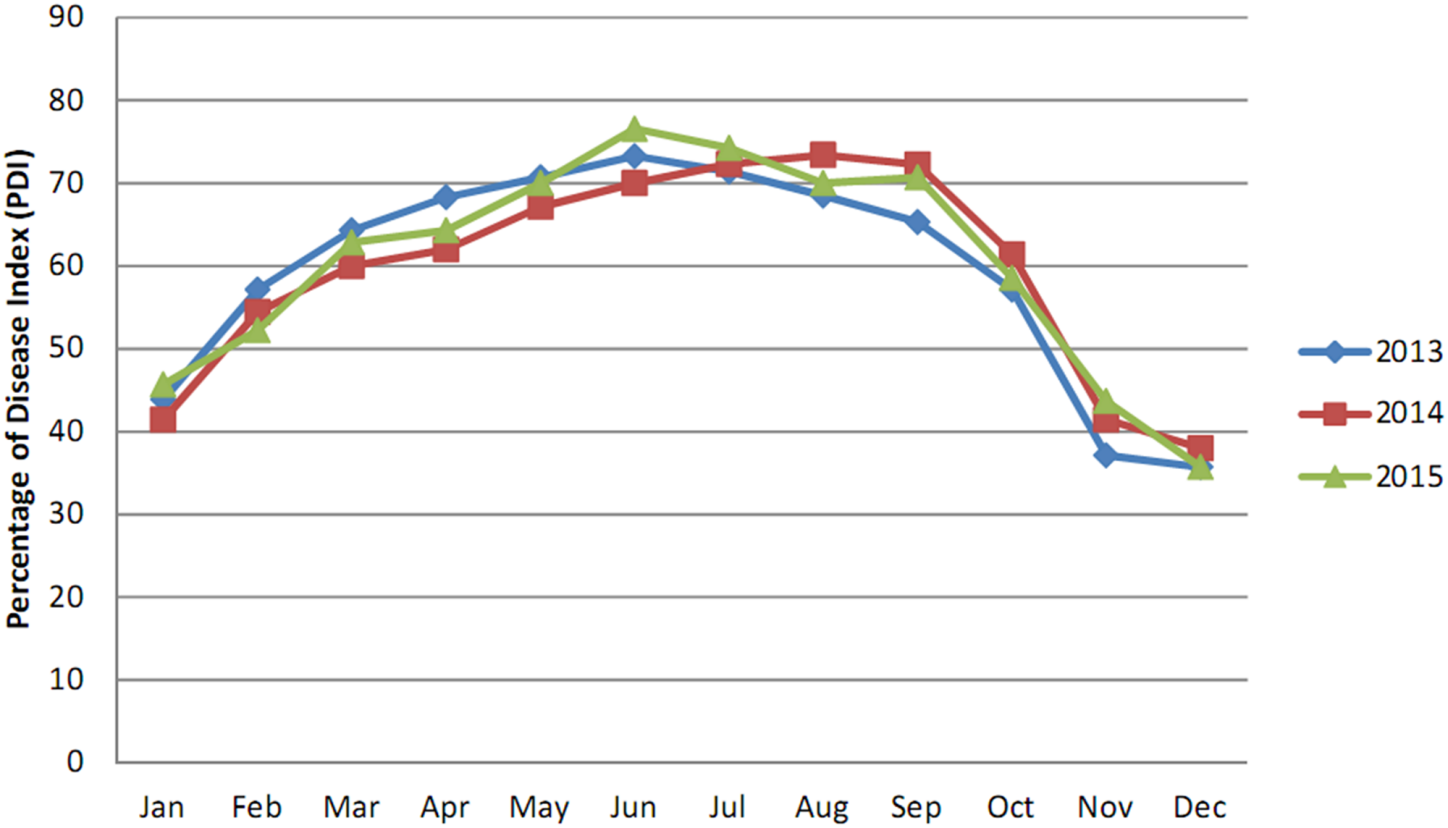
Monthly disease progression curve of *Aloe* leaf spot disease from January, -2013 to December,-2015 at the Agri Horticultural Society, Alipore.

According to the data in Table 1, the percentage of disease index (PDI) for *Aloe* leaf spot disease was greatest during May,- 2013 (94.31%) in Nilganj where as during December,-2014, it decreased to 44.31%. At the Agri Horticultural Society of India, Alipore, the effect of the disease was greatest during June,- 2015 (76.57%) and mildest during December, 2013 and December,-2015 (35.71%) (Figs 3 and 4).

**Table 1:** Percentage of disease index (PDI) for leaf spot disease of *Aloe vera* in Nilganjandat the Agri Horticultural Society (AHS) of India, Alipore.

Month	Nilganj			Pool data **SD+/-	AHS, Alipore			Pool data **SD+/-
	2013	2014	2015		2013	2014	2015	
**Jan**	57.27(49.14)	55.68(48.22)	54.54(47.58)	55.83(48.33) 0.640*	43.87(41.44)	41.42(39.82)	45.71(42.53)	43.66(41.32) 1.114*
**Feb**	64.09(53.13)	59.09(50.18)	61.36(51.53)	61.51(51.65) 1.206*	57.14(49.08)	54.42(47.52)	52.25(46.26)	54.60(47.64) 1.156*
**Mar**	79.77(63.22)	72.50(58.37)	75.90(60.60)	76.05(60.67) 1.983*	64.28(53.25)	60.00(50.77)	62.85(52.42)	62.37(52.12) 1.031*
**Apr**	90.45(71.95)	85.90(67.94)	88.18(69.82)	88.17(69.82) 1.640*	68.28(55.67)	62.00(51.94)	64.28(53.25)	64.85(53.61) 1.545*
**May**	94.31(76.19)	90.75(70.24)	89.45(71.00)	91.50(73.05) 2.705*	70.71(57.23)	67.14(55.00)	70.00(56.79)	69.28(56.29) 0.965*
**Jun**	89.77(71.23)	91.59(73.05)	91.69(73.15)	91.01(72.54) 0.884*	73.28(58.82)	70.00(56.79)	76.57(61.00)	73.28(58.82) 1.719*
**Jul**	82.95(65.57)	79.54(63.08)	79.54(63.08)	80.67(63.87) 1.174*	71.42(57.67)	72.30(58.24)	74.28(59.47)	72.66(58.44) 0.751*
**Aug**	76.5(61.00)	75.65(60.40)	78.40(62.31)	76.85(61.21) 0.797*	68.48(55.80)	73.42(58.95)	70.00(56.79)	70.63(57.17) 1.315*
**Sep**	73.86(59.21)	76.81(61.21)	77.80(61.89)	76.15(60.73) 1.138*	65.28(53.85)	72.28(58.18)	70.71(57.23)	69.42(56.42) 1.858*
**Oct**	63.63(52.71)	68.18(55.61)	65.00(53.73)	65.60(54.09) 1.203*	57.14(49.08)	61.42(51.59)	58.57(49.89)	59.04(50.18) 1.045*
**Nov**	54.54(47.58)	55.68(48.22)	53.63(47.06)	54.61(47.64) 0.474*	37.14(37.52)	41.42(40.05)	43.71(41.38)	40.75(39.64) 1.601*
**Dec**	45.77(42.53)	44.31(41.73)	47.72(43.68)	45.93(42.65) 0.800*	35.71(36.69)	38.00(38.06)	35.71(36.69)	36.47(37.11) 0.646*
CD (p 0.05) SE ±				12.60 6.08	CD (p 0.05) SE ±			12.64 6.10

*Values within parentheses denote the angular transformation value.

** Indicates SD± value.

**Fig 5:**
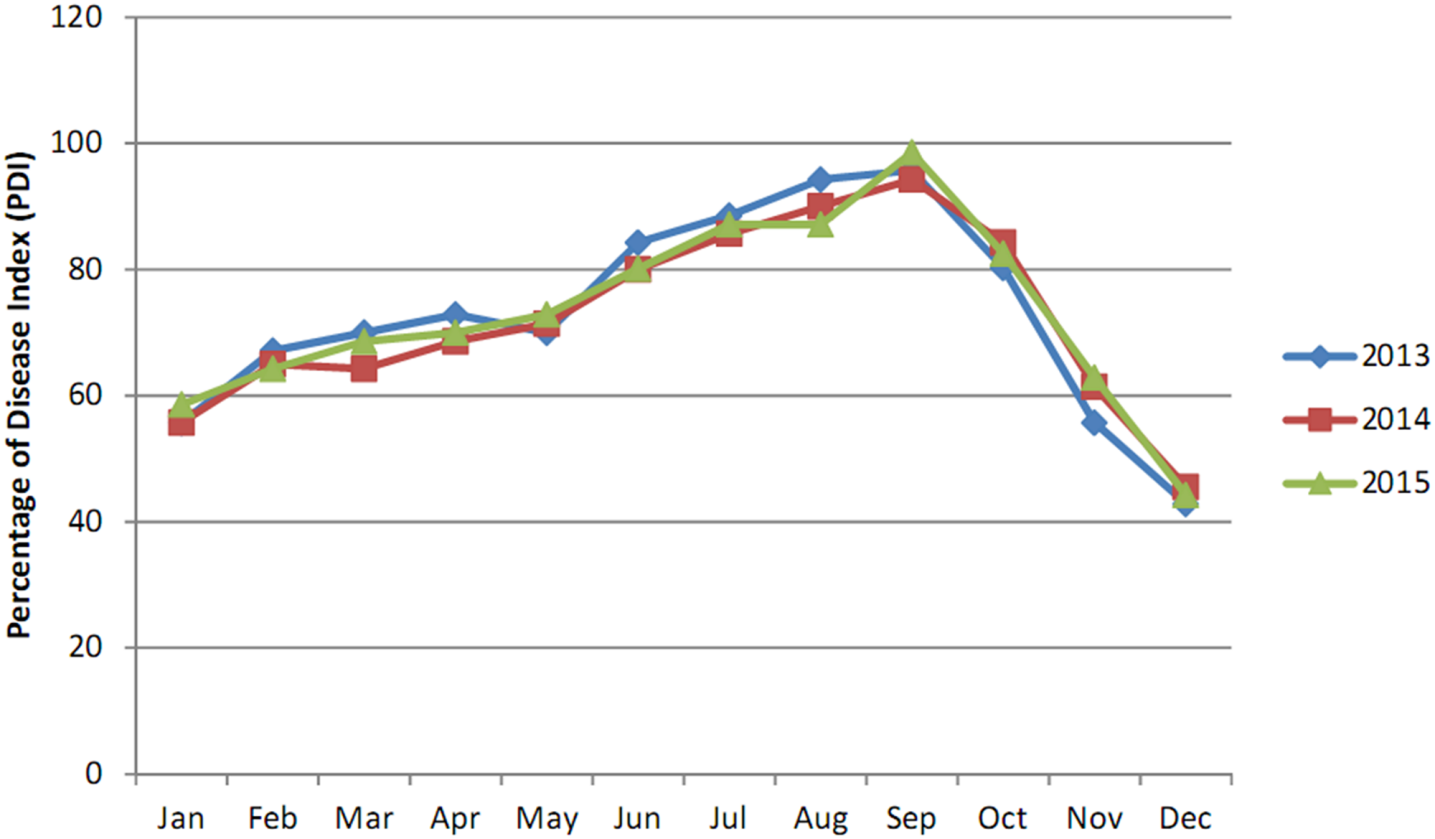
Monthly disease progression curve of *Aloe* leaf spot disease from January,-2013 to December, 2015 at the State Pharmacopical Laboratory and Pharmacy for Medicine, Nadia.

**Fig 6:**
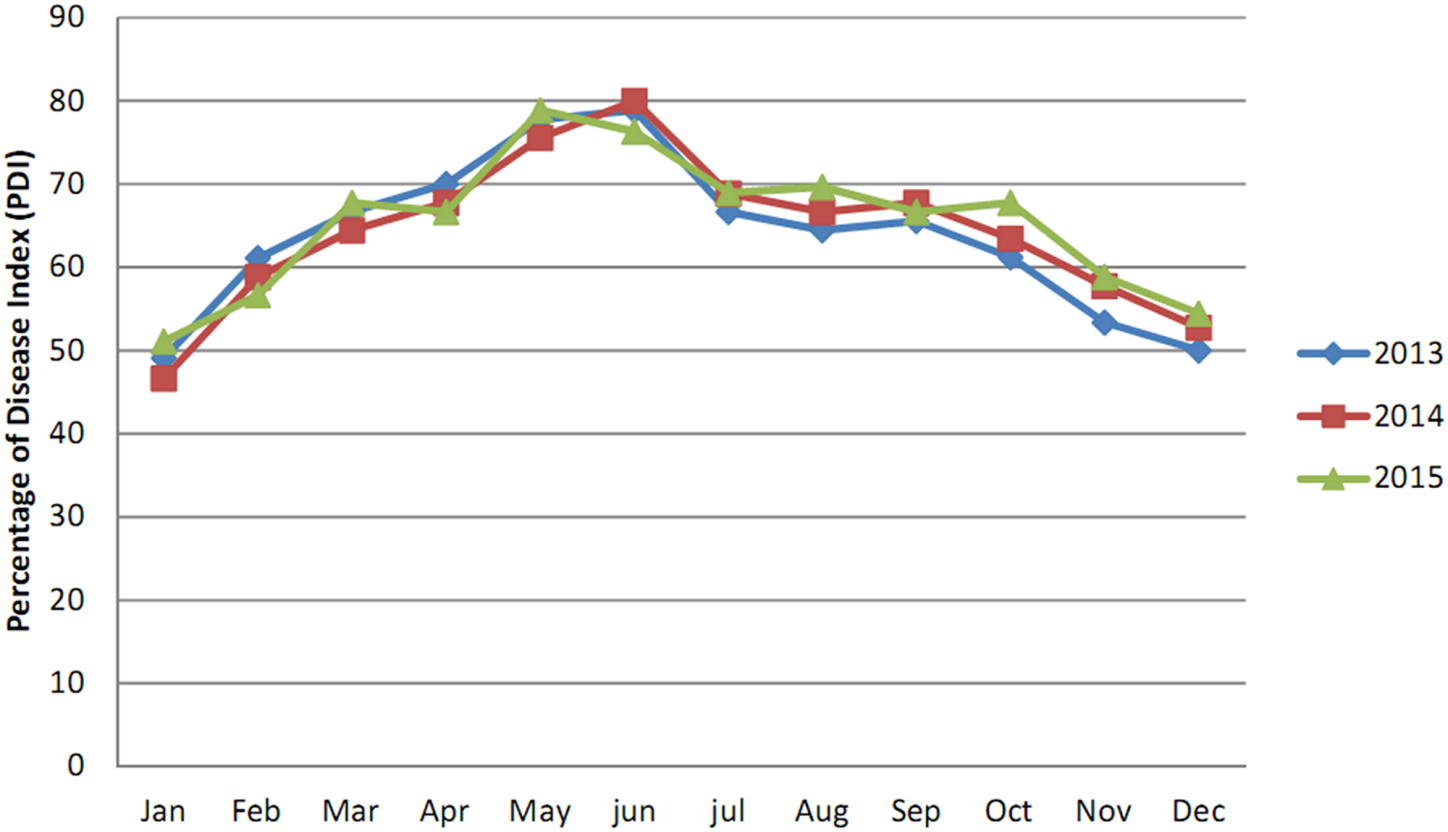
Monthly disease progression curve of *Aloe* leaf spot disease from January, -2013 to December, -2015 in Narendrapur.

The data presented in Table 2 reveal that the PDI for *Aloe* leaf spot disease reached its peak during, May2015 (98.57%) in the State Pharmacopoeial Laboratory and Pharmacy for Medicine, Nadia where as during, December2013 it decreased to 42.85%. In Narendrapur the effect of the disease was highest during June;-2014 (80.00%) and lowest during January; 2014 and December;-2015 (46.66%) (Figs 5 and 6).

**Table 2:** Percentage of disease index (PDI) for leaf spot disease of *Aloe vera* at the State Pharmacopoeial Laboratory and Pharmacy for Medicine, Nadia (State Pharma), and Narendrapur.

Months	State Pharma, Nadia				Narendrapur			
	2013	2014	2015	Pool data **SD+/-	2013	2014	2015	Pool data **SD+/-
**Jan**	55.71(48.27)	55.71(48.27)	58.57(49.89)	56.66(48.79) 0.763*	49.11(44.46)	46.66(43.05)	51.11(45.63)	48.96(44.37) 1.054*
**Feb**	67.14(55.00)	65.00(53.73)	64.28(53.25)	65.47(53.97) 0.738*	61.11(51.41)	58.88(50.07)	56.66(48.79)	58.88(50.07) 1.069*
**Mar**	70.00(56.79)	64.28(53.25)	68.57(55.86)	67.61(55.30) 1.498*	66.66(54.70)	64.44(53.37)	67.77(55.37)	66.29(54.45) 0.831*
**Apr**	72.85(58.56)	68.57(55.86)	70.00(56.79)	70.47(57.04) 1.120*	70.00(55.79)	67.77(55.37)	66.66(54.70)	68.14(55.61) 0.553*
**May**	70.00(56.79)	71.42(57.67)	72.85(58.56)	71.42(57.67) 0.722*	77.77(61.82)	75.55(60.33)	78.88(62.58)	77.40(61.62) 0.935*
**Jun**	84.28(66.58)	80.00(63.44)	80.14(63.51)	81.47(64.45) 1.465*	78.88(62.58)	80.00(63.44)	76.33(60.87)	78.40(62.31) 1.068*
**Jul**	88.57(70.18)	85.71(67.78)	87.14(68.95)	87.14(68.95) 0.980*	66.66(54.70)	68.88(56.04)	68.98(56.11)	68.17(55.61) 0.648*
**Aug**	94.28(76.06)	90.00(71.56)	87.14(68.95)	90.47(71.95) 2.946*	64.44(53.37)	66.66(54.70)	69.66(56.54)	66.92(54.88) 1.299*
**Sep**	95.71(78.03)	94.28(76.06)	98.57(82.96)	96.18(78.61) 2.930*	65.55(54.03)	67.77(55.37)	66.66(54.70)	66.66(54.70) 0.547*
**Oct**	80.28(63.58)	84.28(66.58)	82.57(65.27)	82.37(65.12) 1.228*	61.20(51.47)	63.44(52.77)	67.77(55.37)	64.13(53.19) 1.621*
**Nov**	55.71(48.27)	61.42(51.59)	62.85(52.42)	59.99(50.71) 1.793*	53.33(46.89)	57.77(49.43)	58.88(50.07)	56.66(48.79) 1.373*
**Dec**	42.85(40.86)	45.50(42.42)	44.28(41.67)	44.21(41.67) 0.637*	50.00(45.00)	52.77(46.55)	54.44(47.52)	52.40(46.38) 1.038*
CD (p 0.05) SE±				15.56 7.52	CD (p 0.05) SE±			9.72 4.69

*Values within parentheses denote the angular transformation value.

** Indicates SD± value.

This study indicated that the maximum disease intensity was occurred between April and September in all four studied areas. This result was probably due to high temperature and moisture as this pathogen is mostly active in wet seasons and in areas with relatively high rainfall [21].

Similarly in a survey conducted on fungal diseases of medicinal plants in Maharashtra State, scientists observed leaf spot disease of *Aloe vera* in the Osmanabad district of Maharashtra [7]. They reported that three pathogens were associated with the disease, namely, *Alternaria alternata*, *A*. *tenuissima* and *Fusarium* sp. According to the authors, the disease was found during the rainy season and winter but absent in summer. However, in this survey, occurrence of the disease was observed throughout the year. This may be due to differences in geographical and climatic factors among the survey areas.

### *In vitro* control of the pathogen

**Fig 7:**
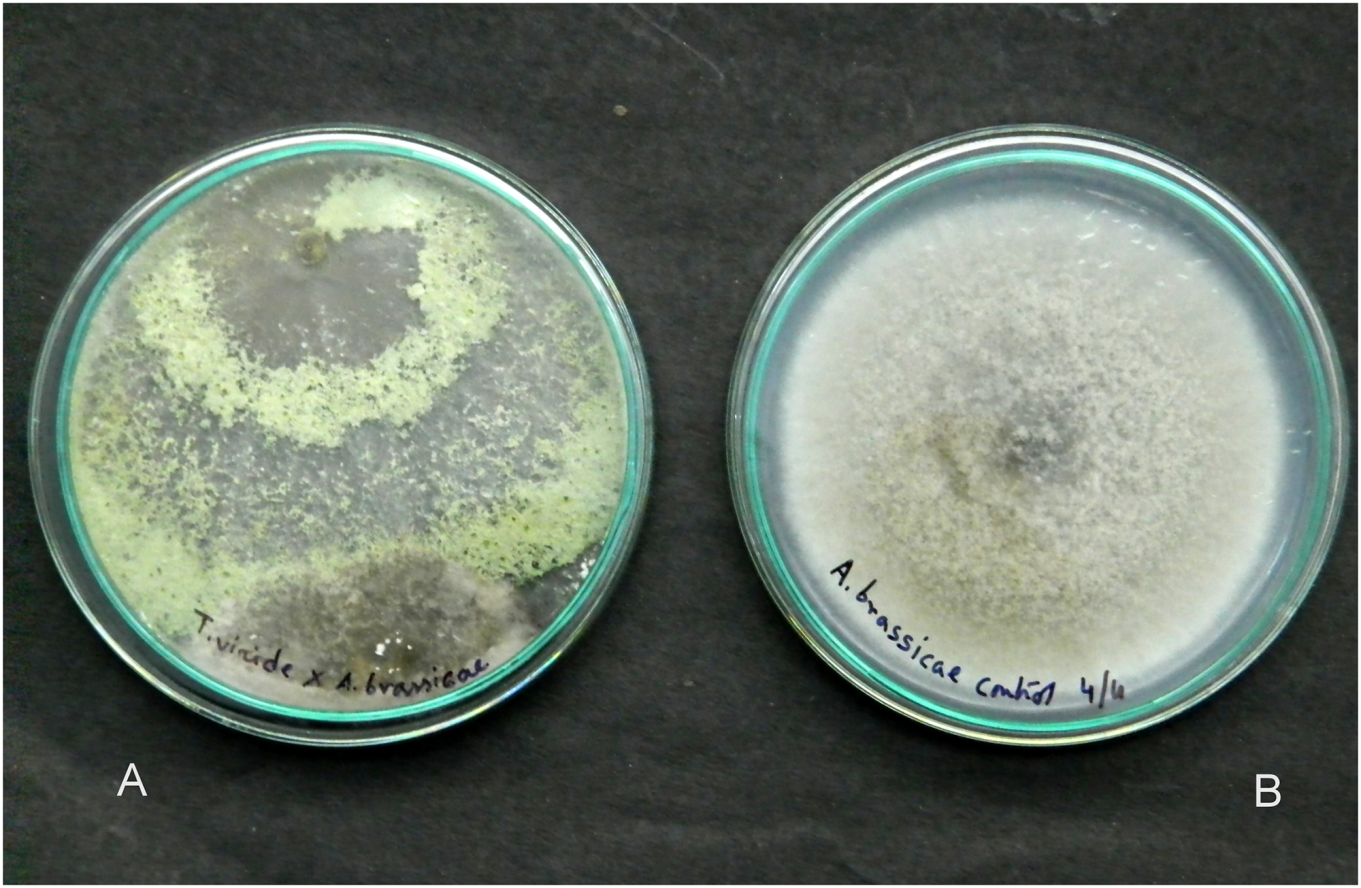
Colony of *A*. *brassicae* completely overgrown by the mycelial growth of *T*. *asperellum* (A); and, control plate of *A*. *brassicae* (B).

**Fig 8:**
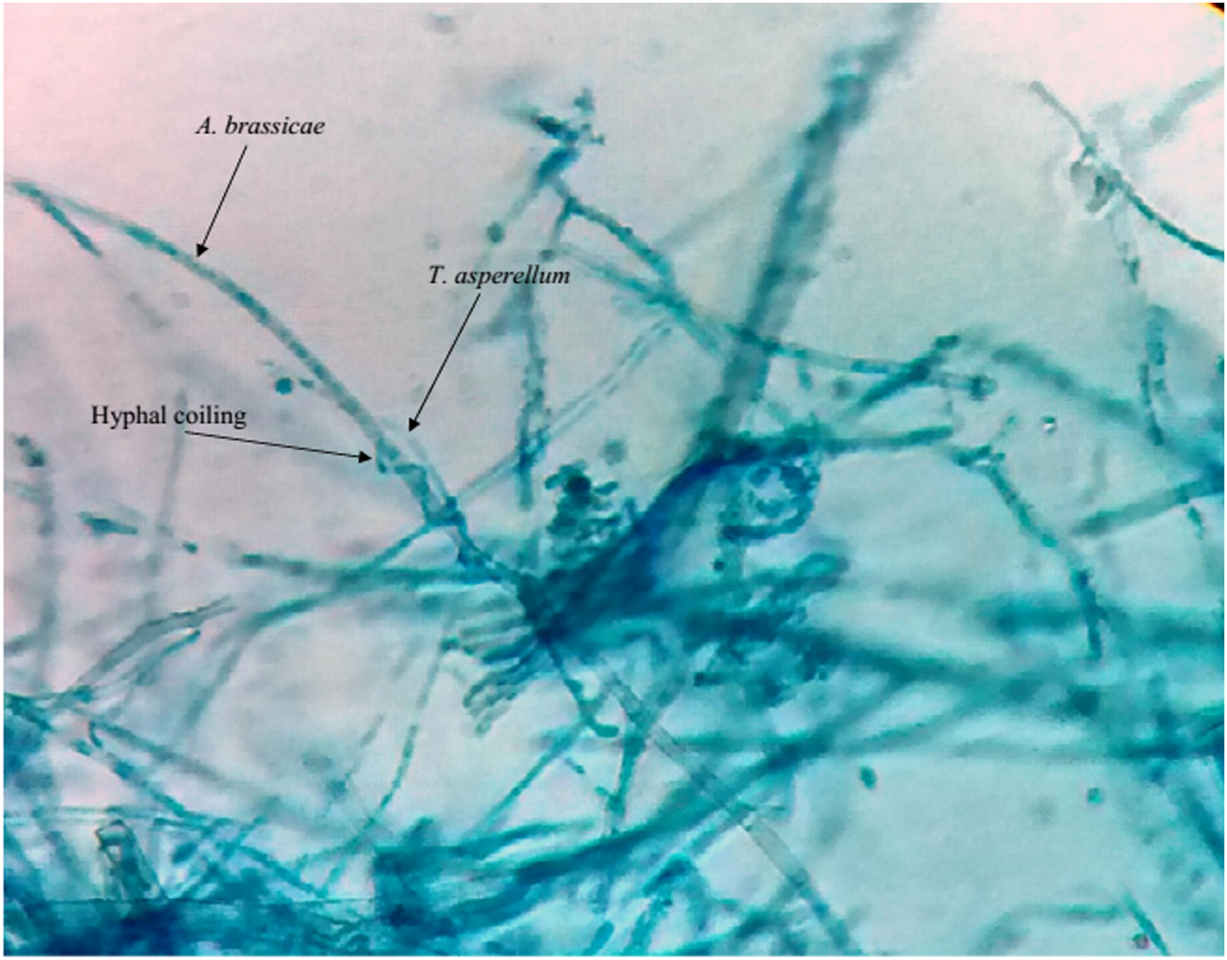
Hyphae of *T*. *asperellum* coils over the hyphae of *A*. *brassicae* (450X).

**Fig 9:**
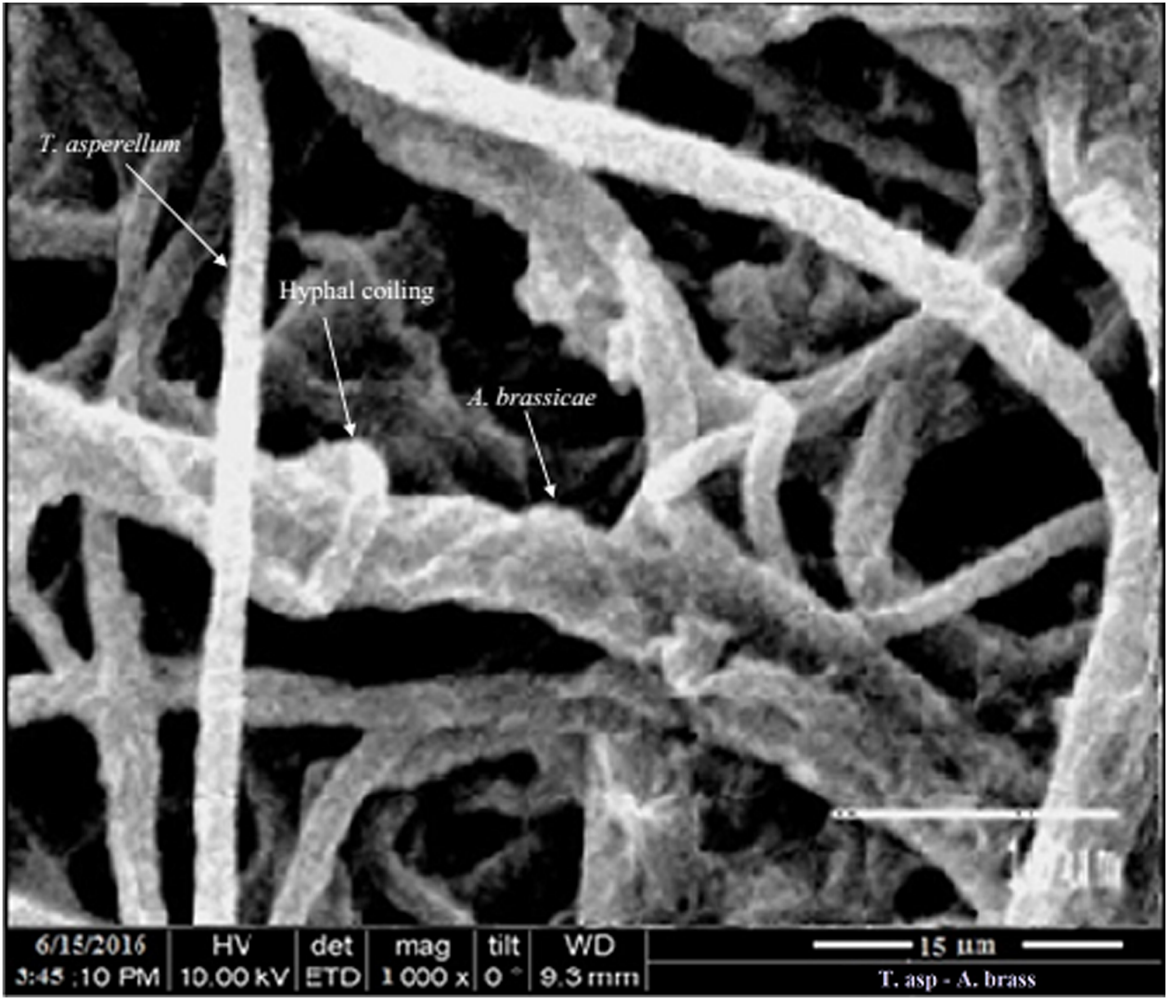
Scanning electron microscopic view of the hyphal coiling of the hyperparasitic biocontrol agent *T*. *asperellum* over the pathogen *A*. *brassicae*.

The pathogen *Alternaria brassicae*, isolated from *Aloe vera* was treated with fungal antagonists such as *Trichoderma viride*, *T*. *harzianum*, *T*. *asperellum*, *T*. *longibrachiatum* and *Beauveria bassiana*. The results presented in Table 3 reveal that the antagonist *T*. *asperellum* was recorded with a maximum percentage of inhibition of radial growth (PIRG) of 70.39% through non volatile metabolites (Fig 7) followed by *T*. *viride* (67.22%), *T*. *harzianum* (65.92%), *T*. *longibrachiatum* (64.80%) and *Beauveria bassiana* (64.61%) in a dual-culture-method. Our compound microscopy (Fig 8) and scanning electron microscopy (SEM) studies (Fig 9) showed features such as hyphal coiling, cellular distortion, and vacuolation that confirmed the hyperparasitism of *Trichoderma* over *A*. *brassicae*.

**Table 3:** Non volatile effects of the fungal biocontrol agents on the radial growth of *Alternaria brassicae*.

BCA	Average radial growth (cm) of pathogen	Percentage of inhibition of radial growth (PIRG) of antagonistic fungi over *A*. *brassicae* (after 7 days)
*T*. *asperellum*	2.65♦	70.39 a** (56.98)#
*T*. *harzianum*	3.05	65.92 b (54.27)
*T*. *viride*	2.92	67.22 ab (55.06)
*T*. *longibrachiatum*	3.15	64.80 b (53.61)
*Beauveria bassiana*	3.16	64.61 b (53.49)
Control (untreated)	8.95	
CD (p = 0.05) 1.876 SE ± 0.676		

^♦^For each set of experiments 3 replicates were performed.

**Note: In the same column the same letter indicates that values statistically the same while different letters indicate that they are significantly different. Mean values followed by a different letter indicate significant differences (*P* = 0.05) according to Duncan’s multiple range test.

^#^values within parentheses denote the angular transformation value.

**Fig 10:**
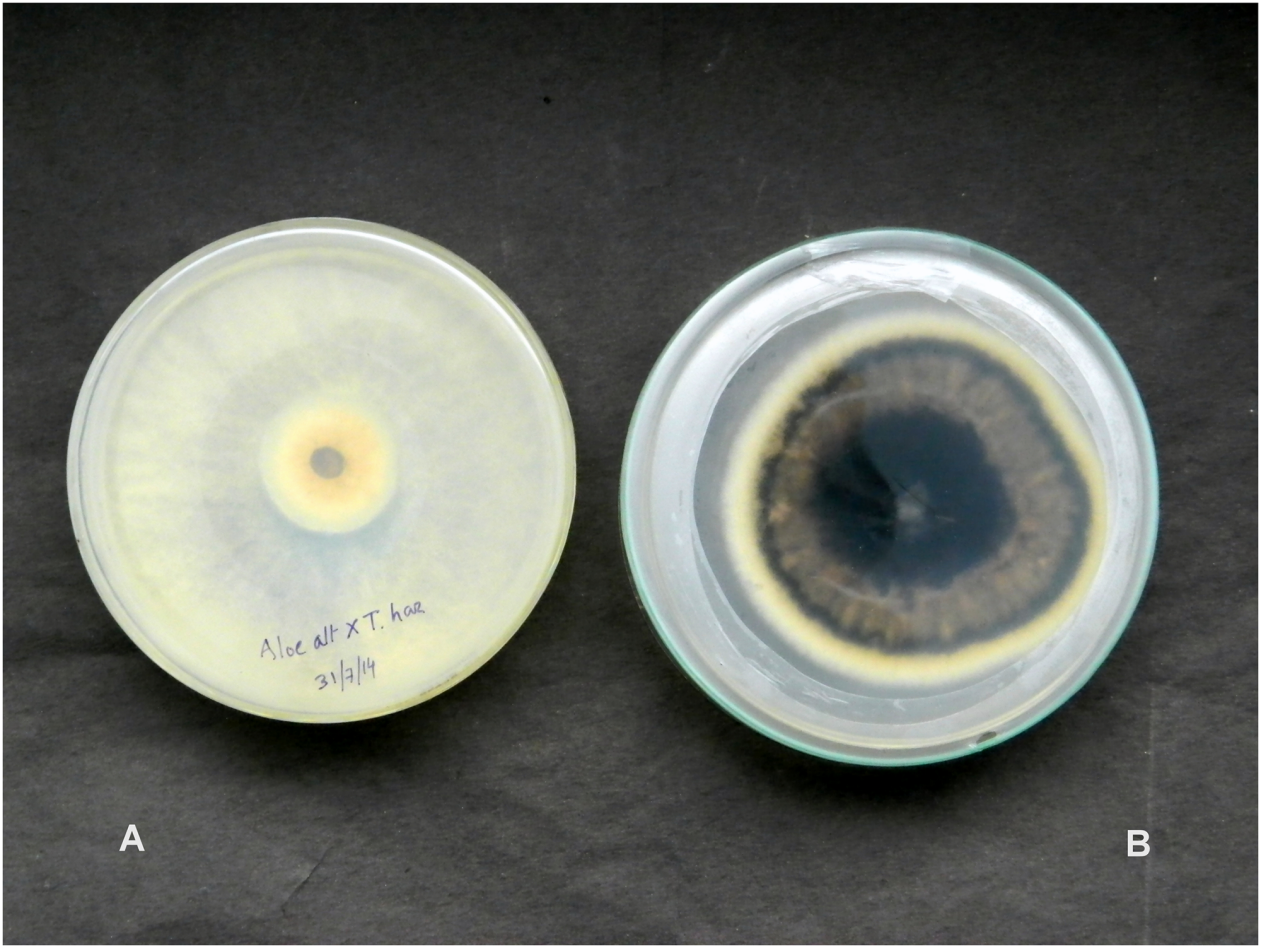
(A) *A*. *brassicae* treated with *T*. *asperellum* (B) Control.

The data presented in Table 4 revealed that the antagonist *T*. *asperellum* was recorded with maximum PIRG of 72.17% through volatile metabolites (Fig 10) followed by *T*. *longibrachiatum* (65.32%), *T*. *harzianum* (63.31%), *T*. *viride* (59.47%) and *B*. *bassiana* (33.46%).

**Table 4:** Volatile effect of the fungal biocontrol agents on radial growth of *Alternaria brassicae*.

BCA	Average radial growth (cm)	Percentage of Inhibition of Radial growth (PIRG) of antagonistic fungi over *A*. *brassicae* (after 7 days)
*T*. *asperellum*	2.30♦	72.17 (58.12)# a**
*T*. *harzianum*	3.03	63.31 (52.71)b
*T*. *longibrachiatum*	2.86	65.32 (53.91) b
*T*. *viride*	3.35	59.47 (50.42) c
*Beauveria bassiana*	5.50	33.46 (35.30)d
Control (untreated)	8.26	
CD (p = 0.05) 1.717 SE ± 0.618		

^♦^For each set of experiment 3 replicates were performed.

**Note: In the same column, the same letter indicates that the values are statistically equivalent, while different letters indicate that they are significantly different. Mean values followed by different letters indicate significant differences (*P* = 0.05) according to Duncan’s multiple range test.

^#^Values within parentheses denote the angular transformation value.

### Detection of volatile substances

**Fig 11:**
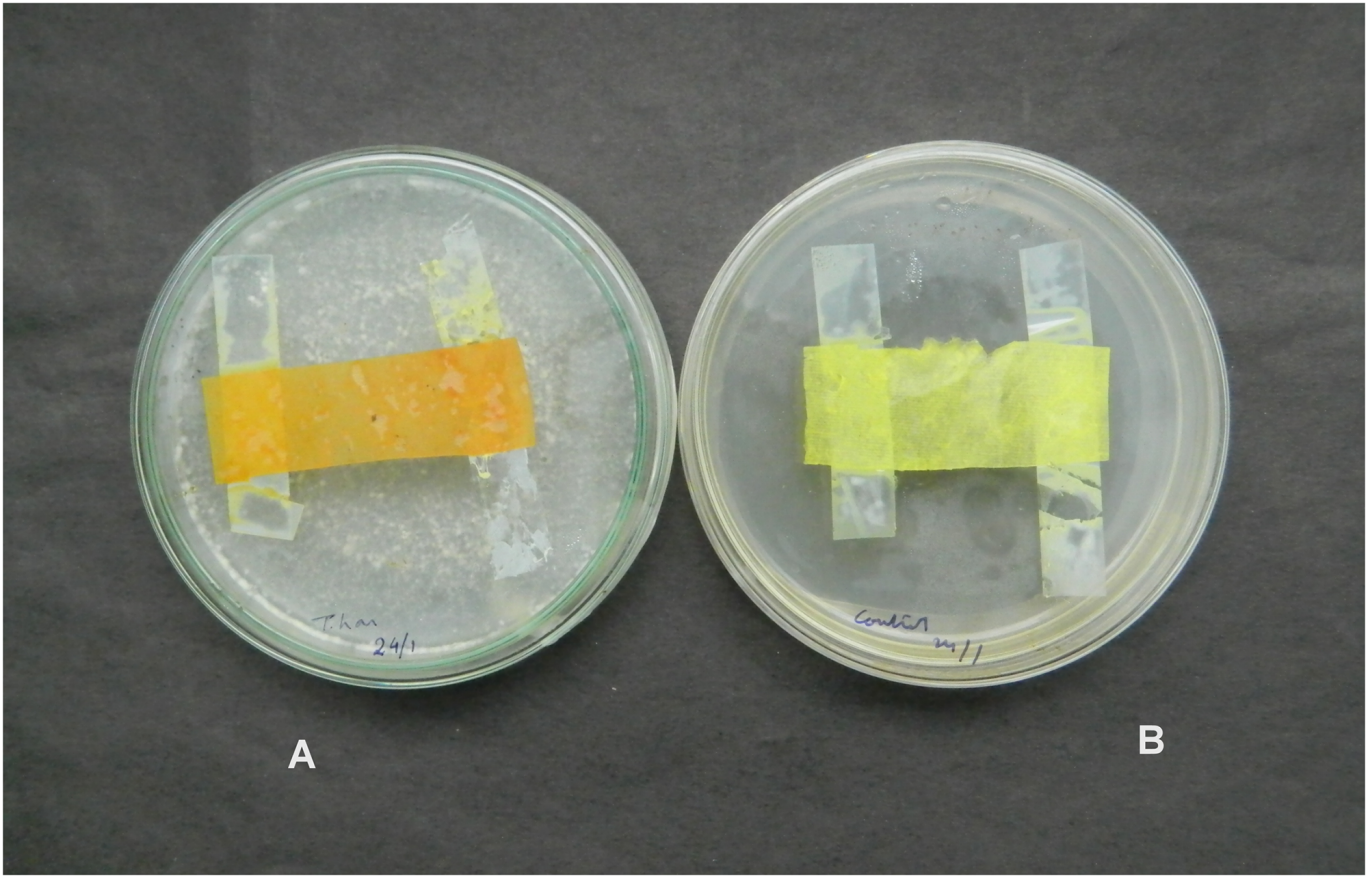
(A) Plate inoculated with *T*. *harzianum*. (B) Control.

**Fig 12:**
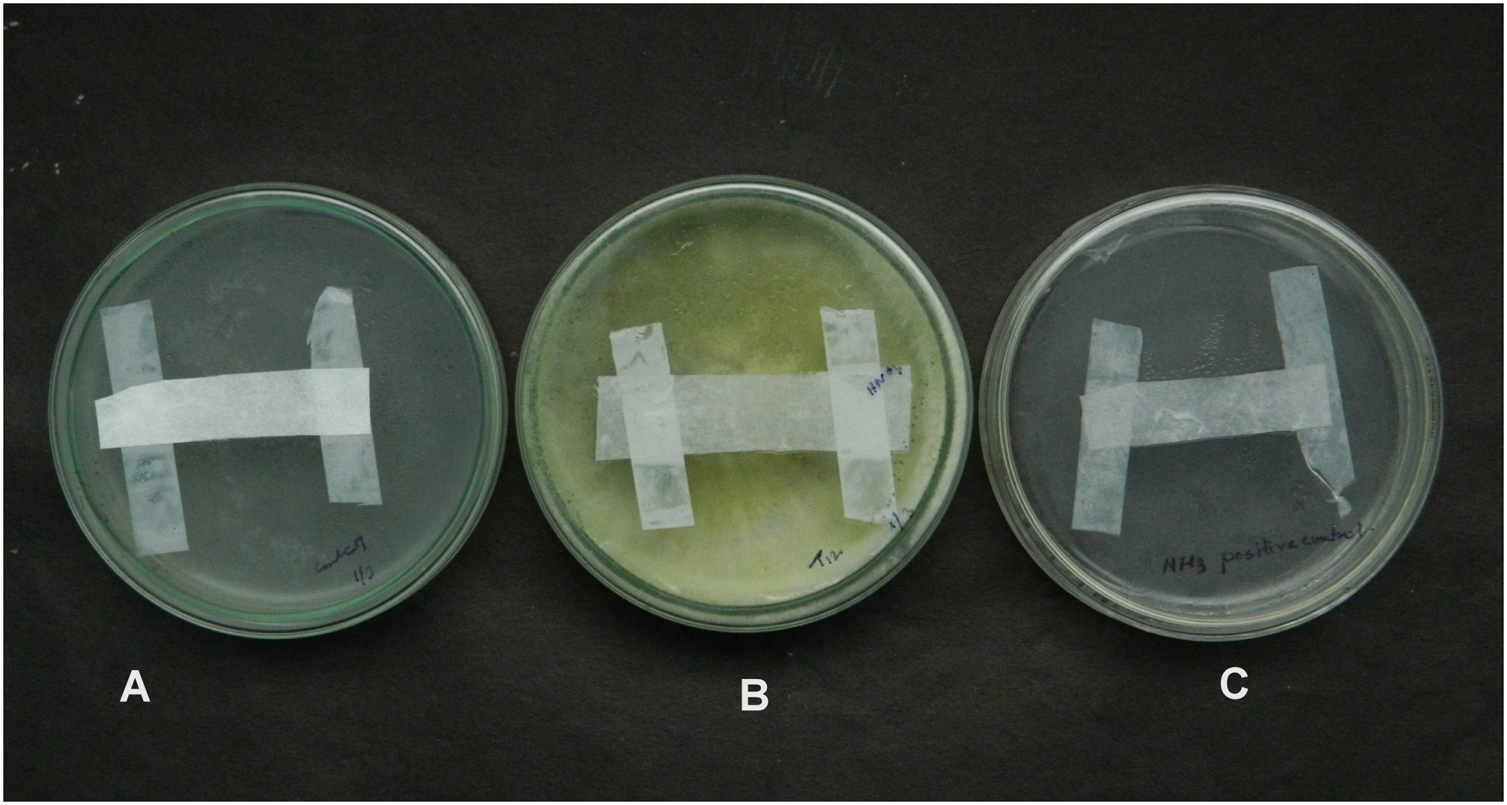
(A) Negative control. (B) Plate inoculated with *T*. *asperellum*. (C) Positive control.

The data presented in Table 5 show that among the five fungal antagonists, only *T*. *harzianum* (Fig 11) produced HCN, where as *T*. *viride*, *T*. *asperellum*, *T*. *longibrachiatum* and *B*. *bassiana* were unable to produce HCN. The role of HCN (hydrogen cyanide) production by biocontrol agents in the biological control of pathogens has been reported by many authors [22] and a positive correlation between HCN production *in vitro* and plant protection has been reported [23]. The direct inhibition of fungi by HCN was thought to be one of the main mechanisms of action. In addition to the biocontrol efficacy of HCN production in rhizosphere numerous reports are also available on the stimulation of root length and root hair germination in tobacco plants[24]. The bacterium *Pseudomonas fragi* CS11RH1 (MTCC8984), produces hydrogen cyanide (HCN), and wheat seeds bacterized with this isolate showed significant increases in the percent germination, rate of germination, plant biomass and nutrient uptake as seedlings. On the other hand, among the five fungal antagonists, four were recorded to produce ammonia (NH_3_) although the production of ammonia was more evident for *T*. *harzianum* (Fig 12), *T*. *viride*, *T*. *longibrachiatum* and *T*. *asperellum* than for *Beauveria bassiana*. The production of ammonia gas as an important biocontrol tool was also characterized in the interaction between the nitrogen fixing bacterium *Enterobacter cloacae* and pathogens such as *Pythium ultimum* and *Rhizoctonia solani* [25]. The liberated volatile substances secreted by biocontrol agents accumulate in the soil where they limit the growth and germination of a wide range of pathogens in agricultural fields, a process that is also favored by mulching techniques [26]. The production of diffusible and volatile metabolites by *Trichoderma* spp. was also reported previously[27; 28; 29]. Meena *et*. *al*.[30] reported that volatile metabolites of *T*. *harzianum* and *T*. *viride* induced 40% and 35% growth inhibition respectively of *Alternaria alternata*,. They further analyzed volatile compounds produced by- *Trichoderma* spp. Through the GC-MS technique and abundance of glacial acetic acid (45.32%) and propyl-benzene (41.75%) were reported as distinguishable antifungal volatile metabolites.

**Table 5:** Production of volatile substances by biocontrol agents.

Fungal antagonist	HCN Production	NH_3_ Production
*T*. *harzianum*	+	+
*T*. *viride*	-	+
*T*. *asperellum*	-	+
*T*. *longibrachiatum*	-	+
*Beauveria bassiana*	-	+/-

(+) indicates the production of volatile substances, and (–) indicates no production of volatile substances. For each set of experiments 3 replicateswereperformed.

Parasitism of fungi particularly mycoparasitism is a special mode of existence for many species. Since the discovery that *Trichoderma* has great potential for biocontrol [31], many researchers working with *Trichoderma* noticed that hyphae of the antagonists parasitized hyphae of other fungi *‘in vitro’* and brought about several morphological changes such as coiling, haustorium formation, disorganization of host cell contents and penetration of the host [32]. Classical evidence of the mycoparasitic activity of *Trichoderma* through phase contrast and electron microscopywas provided previously through advanced microscopic studies [33,34]. Our compound microscopy (Fig 8) and SEM studies (Fig 9) showed hyphal coiling, cellular distortion, and vacuolation, among other features and these findings are supported by previous studies [31,32,33]. *T*. *viride* and *T*. *harzianum* [35,36,37,38,39,40,41] showed the potential of *in vitro* antagonistic activity against the important plant pathogens such as *Sclerotinia sclerotiorum*, *Pyricularia oryzae*, *Bipolaris oryzae*, *Alternaria solani*. *A*. *alternata*. *Phytophthora colocasiae*, *P*. *parasitica* var. *piperina*, *Pythium aphanidermatum* and *Colletotrichum gloeosporioides*. Antibiosis, mycoparasitism and competition are the main mechanisms of biological control [42,43]. The production of diffusible and volatile metabolites by *Trichoderma* spp. was reported previously [27,28,29]. *Trichoderma* strains exert biocontrol against fungal phytopathogens either indirectly, by competing for nutrients and space, modifying the environmental conditions, or promoting plant growth and defense mechanisms and antibiosis, or directly, by mechanisms such as hyperparasitism. The activation of each mechanism implies the production of specific compounds and metabolites such as plant growth factors, hydrolytic enzymes, siderophores, antibiotics and carbon-nitrogen permeases [44]. Pathogen inhibition in co-cultures begins soon after contact with the antagonist [45]. *Trichoderma* spp. adhere exactly on other fungal pathogenic hyphae, where they coil around them and degrade the cell walls. This action of parasitism restricts the development and activity of pathogenic fungi. Additionally, or together with mycoparasitism, some *Trichoderma* species release antibiotics [46]. While analyzing the biocontrol—plant pathogen interaction, *Trichoderma virens* spores or cell-free culture filtrate was reported to regulate the growth and the defense responses of tomato against Fusarium wilt disease of tomato (C.O.- *Fusarium oxysporum* f. sp. *lycopersici*) through induction of higher levels of salicylic acid and jasmonic acid in the host; those phytohormones in turn served as inducers of various pathogenesis related proteins, resulting in significantly lower disease incidence [18]. Plant immunization with rhizospheric fungi such as *Trichoderma harzianum* resulted in resistance of tomato against bacterial wilt disease through increased production of defense-related enzymes such as peroxidase, phenylalanine ammonia lyase, β-1,3-glucanase along with early emergence and increased vigor of the fruit [47]. Nagaraju *et*. *al*.[48] evaluated the efficacy of seven different rhizospheric fungi from various regions of Southern India for plant growth promotion and disease resistance against sunflower downy mildew disease(C.O.- *Plasmopara halstedii*). According to those authors, priming seed with a conidial suspension of PGPF at 1 × 10^8^CFU mL^-1^ significantly increased seed germination and seedling vigor, as well as reducing downy mildew disease severity by 61%, compared to the untreated control. *T*. *asperellum* was recorded to show prolific activity in controlling *Fusarium* wilt in chickpea (75.25% and 67.15% of crop protection in two consecutive years) and to act as an excellent growth promoting factor in a mini pot trial. The field application of *T*. *hamatum* and *T*. *koningii* can increase crop productivity up to 300% [49]. Similar results were also reported by some other researchers in green houses for seed treatment with *Trichoderma* spores [50]. The activity of biocontrol agents could also reduce the soil concentrations of substances that inhibit to plant growth [51]. Thus, the plant growth promotion may be due to the production of plant hormones or the increased uptake of nutrients by the plant [52], control of other pathogens at subinfectious levels and/or strengthening plants own defense mechanisms[53].

### Effect of fungicides on the radial growth of the pathogen

The data presented in Table 6 demonstrate that the most potent biocontrol agent *T*. *asperellum* showed the highest PIRG against *A*. *brassicae in vitro* (70.39%), which was highly comparable to 2% blitox-50 (73.92%) and much better than 2% bavistin (59.77%). Additionally, *T*. *harzianum* (65.91%) and *T*. *viride* (67.22%) also exerted significant *in vitro* control over the leaf spot pathogen *A*. *brassicae*. More importantly bavistin and blitox-50 are toxic to the environment and are carcinogenic. Therefore, in terms of environmental hazards, application of *T*. *asperellum* not only provided a more or less comparable result with 2% blitox-50 but also provided a much better result than 2% bavistin in *in vitro* conditions.

**Table 6:** *In vitro* comparison between applications of fungicides and of the most potent biocontrol agents.

Treatment	Average radial growth (cm) of *A*. *brassicae*	Average PIRG of *A*. *brassicae*
Bavistin (2% conc.)	3.60	59.77 (50.59) c
Blitox-50 (2% conc.)	2.33	73.92 (59.28) a
*T*. *asperellum*	2.65	70.39 (56.98) a
*T*. *harzianum*	3.05	65.91 (54.27) b
*T*. *viride*	2.92	67.22 (55.06) b
Control (untreated)	8.95	
CD (p = 0.05) 2.068 SE ± 0.745		

For each set of experiments, 3 replicates were performed. Note: In the same column the same letter indicates that values are statistically equivalent, while different letters indicate that they are significantly different. Mean values followed by different letters indicate significant differences (*P* = 0.05) according to Duncan’s multiple range test. Data presented in the parentheses represent the angular transformation value

### Field trial for the *in vivo* study

**Fig 13:**
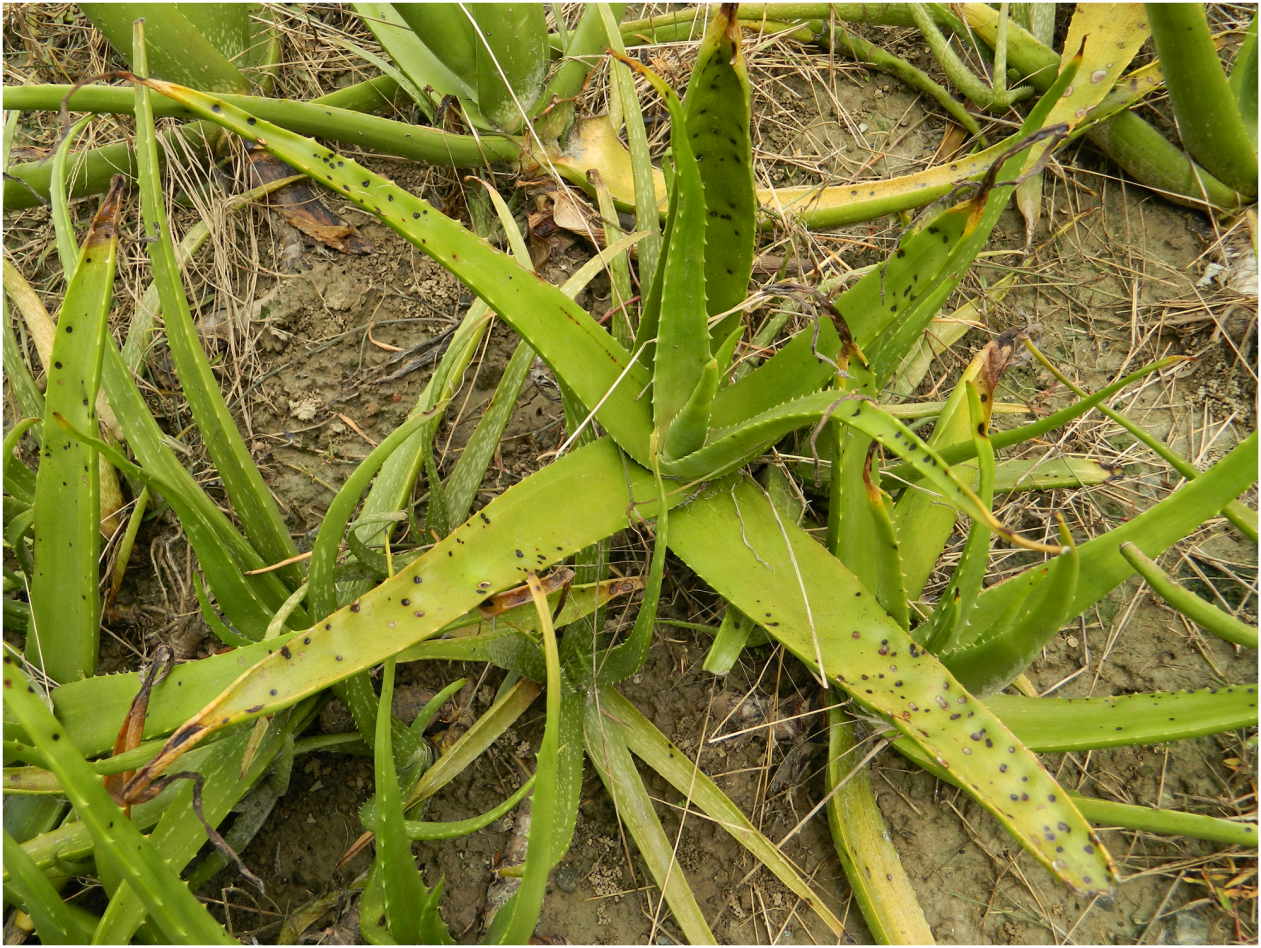
Untreated saplings of *Aloe vera* showing many leaf spots.

**Fig 14:**
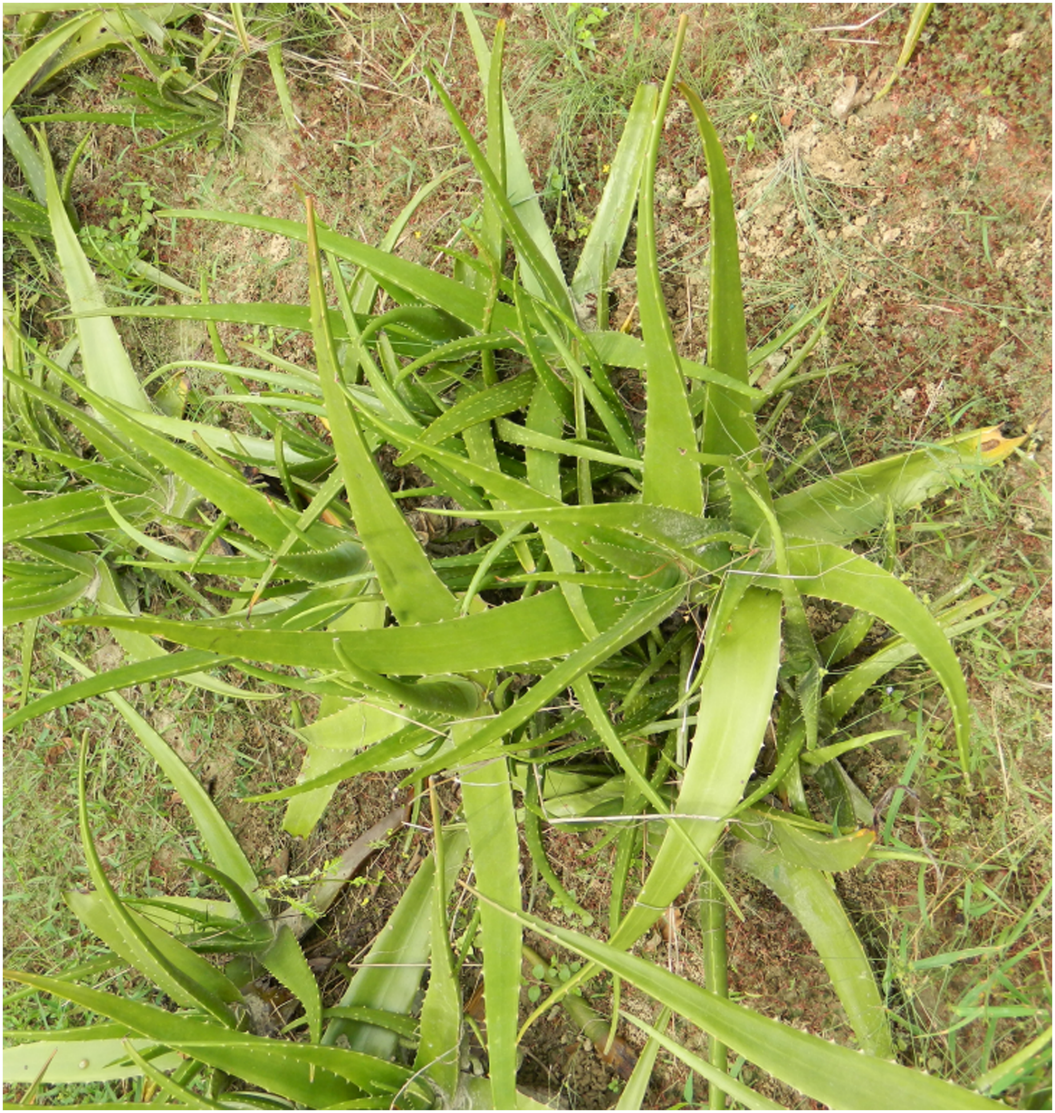
*Aloe vera* treated with a spore suspension of *T*. *asperellum* (10^8^ spores/mL) showing a negligible number of leaf spots.

Pool data presented in Table 7 revealed that based on analysis of three years (2013, 2014 and 2015) of data, the percentage of disease index (PDI) values in control plots were much higher (73.33%) (Fig 13) than those in their treated counterparts (Fig 14). Plots treated with *T*. *asperellum* only showed in 24.00% PDI (Fig 14). Statistically similar results were obtained in plots treated with *T*. *viride* (25.33%) and *T*. *harzianum* (29.33%). Therefore, there was a significant decrease in terms of pool data of PDI as a result of the application of fungal biocontrol agents *T*. *asperellum*, *T*. *viride* or *T*. *harzianum* in spore suspension at a rate of 10^8^ spores / mL. This is the first attempt to apply *T*. *asperellum*, *T*. *viride* or *T*. *harzianum* (10^8^ spores/ mL) as biocontrol agents at the standardized rate of 2.5 L / plot (36 sq ft) against this epidemic leaf spot disease of *Aloe vera* in a comprehensive approach to the ecofriendly management of this disease.

**Table 7:** Field trial of biocontrol agents on *Aloe vera* through mini plot design.

Treatments	2013	2014	2015	Pool data
***T*. *asperellum***	28.00 * (31.95)♦	20.00 (26.56)	24.00 (29.33)	24.00 (29.33) b°
***T*.*harzianum***	28.00 (31.95)	32.00 (34.45)	28.00 (31.95)	29.33 (32.77) b
***T*. *viride***	24.00 (29.33)	28.00 (31.95)	24.00 (29.33)	25.33 (30.20) b
**Untreated**	72.00 (58.05)	68.00 (55.55)	80.00 (63.44)	73.33 (58.89) a
CD (p = 0.05) SE ±				5.86 2.40

^♦^Data within parentheses denotes the angular transformation value.

°Note: In the same column, the same letter indicates that values are statistically equivalent, while different letters indicate that they are significantly different. Mean values followed by different letters indicate significant differences (*P* = 0.05) according to Duncan’s multiple range test.

* For each set of experiments, 3 replicates were performed.

In conclusion, among the biocontrol agents tested by using the dual-culture-plate method, *T*. *asperellum* showed the highest PIRG, 70.39%. The hyperparasitic nature of the biocontrol agents was confirmed by hyphal coiling, as well as cellular changes such as granulation, distortion and vacuolation induced by *T*. *asperellum*, *T*. *harzianum* and *T*. *viride* over *A*. *brassicae*, phenomena that were confirmed through compound microscopy as well as SEM. When analyzing the effect of volatile metabolites on the radial growth of pathogens, *T*. *asperellum* again showed a maximum PIRG of 72.17%. *T*. *asperellum* showed the highest PIRG against *A*. *brassicae in vitro* (70.39%), a value that was quite comparable to that of2% blitox-50 (73.92%) and even better than 2% bavistin (59.77%) treatment for the same pathogen. More importantly bavistin and blitox-50 are toxic to the environment and are carcinogenic. Treating saplings via the dip method (10^8^ spores / mL) and spraying 4 times with a spore suspension standardized at a rate of 2.5 L / plot (36 square feet) is suggested for managing this epidemic leaf spot disease of *Aloe vera* in widespread agricultural fields.

## Material and methods

### Study of disease occurrence and intensity

Four agricultural farmers fields such as i) Nilganj, North 24-Parganas, ii) the State Pharmacopoeial Laboratory and Pharmacy for Medicine, Govt. of W.B., Nadia, iii) the Agri Horticultural Society of India, Alipore and iv) Narendrapur were selected for this purpose. Although specific permissions are not needed, but we have taken the permission as follows-

1. Nilgunj, North 24-Parganas- private farmers field permission has been taken
2. State Pharmacopical Laboratory and Pharmacy for Medicine, Govt. of W.B., Nadia permission from the director has been taken
3. The Agri Horticultural Society of India, Alipore- permission from joint secretary has been taken
4. Narendrapur. private farmers field and permission has taken
5. Moreover, we declare that the field studies did not involved endangered or protected species.

An extensive survey was also carried out in a few selected areas of North 24 Parganas to record the disease occurrence of *Aloe vera* from January 2013 to December 2015. An intensive survey during the same time period was conducted in order to record the percentage of disease index (PDI) by monitoring the cultivation of *Aloe vera* in the 4 selected areas of West Bengal. The fields were visited once per week for the entire study duration to evaluate the PDI. For the study of PDI, three plots from each of the four areas were chosen and 50 plants per plot were randomly selected and tagged. Tagged plants were visited, once per week from January 2013 to December 2015. The disease was rated on the following scale: 0 = 1%, 1 = 10%; 2 = 25%; 3 = 40% 4 = 60%, 5 = 70%, 6 = 90% and 7 = 95%. The PDI was calculated by using the following formula.

$$PDI=\frac{A}{B\times 100}$$

where A = Total number of infected plants; and B = Total number of selected plants.

### Characterization and identification of the pathogen

The isolation, purification and identification of the pathogen were previously conducted phenotypically in our laboratory following published keys; as well as by molecular identification. Genomic DNA of fungi was extracted by the modified CTAB method [54]. Internal transcribed spacer regions (ITS1-5.8S-ITS2 of rDNA) from genomic DNA were amplified by PCR using a DNA amplification reagent kit manual (GeNei) along with the fungus- specific forward primer ITS-1 F (CTTGGTCATTTAGAGGAA-GTAA) [55] and the reverse primer ITS-4 (TCCTCCGCTTATTGATA-TGC)[56]. The sequencing of PCR product was carried out [57] in the Sci Genome Labs Pvt Ltd, Kerala, India; the sequence obtained was submitted to NCBI GenBank and the accession number was obtained. The sequence analysis was carried out using the BLAST bioinformatic tool of NCBI in earlier work from this laboratory [5].

### Antagonistic fungi used

Previously, in our laboratory, four species of *Trichoderma* and one species of *Beauveria* were phenotypically isolated, characterized and identified following published keys [58,59,60] and through DNA extraction [54], PCR amplification of ITS regions using ITS forward [55] and reverse primer [56], and sequencing of the PCR product (ITS1-5.8s-ITS2 of rDNA) [57]. They were published in NCBI GenBank as *Trichoderma asperellum* (GenBank accession no KY966018.1), *Trichoderma viride* (GenBank accession no KY966032.1), *T*. *harzianum* (GenBank accession no KY966015.1), and *B*. *bassiana* (GenBank accession no KM604668.1). *Trichoderma longibrachiatum* was phenotypically characterized, identified and deposited into IMTECH, Chandigarh, India under accession no. MTCC11582).

### Antagonistic potentiality test of isolated antagonistic fungi(dual culture plate method)

Pathogens and antagonists were grown on separate sterilized Petri dishes containing PDA medium. After 5 days, 5-mm discs of young vigorously growing cultures of both pathogens and antagonists were uniformly cut out by using a cork borer and placed on opposite sides of a 9-cm diameter sterilized Petri dish containing 15 mL of PDA and incubated in a BOD incubator at 28± 1°C for 7 days. A plate containing the pathogen served as the control. After 7 days, the radial growth of the pathogen was recorded for each test plate. Antagonistic activity was measured by percentage of inhibition of radial growth (PIRG). The PIRG was calculated with the following formula[61].

$$PIRG=\frac{\left(A-B\right)}{A\times 100}$$

A = Mean diameter of growth in the control and B = Mean diameter of growth in a given test.

#### Effect of the volatile metabolites of antagonistic fungi on the radial growth of pathogens

To investigate the inhibitory effect of volatile components released by the antagonistic fungi or bacteria, the “inverted plate technique” (Dennis and Webster, 1971) was adopted. First, 15 mL of PDA medium was poured into the lower half of a Petridish. Fifteen mL of PDA medium was poured into the upper half of a Petri dish and both halves were allowed to cool and solidify. Next, at the centre of the upper half, a 5-day-old pathogen cultures of 5 mm in diameter was aseptically inoculated, and at the center of the lower half, a 5-mm-diameter 5-day-old culture of an antagonistic fungus was aseptically inoculated. Then, the upper and lower halves of the Petri dish were fixed to each other by paraffin film. In the control set, only the upper half was inoculated. After 7 days the radial growth of the pathogen was measured. The PIRG was calculated with the following formula [61].

$$PIRG=\frac{\left(A-B\right)}{A\times 100}$$

A = Mean diameter of growth in the control and B = Mean diameter of growth in a given treatment.

### Detection of volatile substances

#### Hydrogen cyanide (HCN) production

Production of HCN was qualitatively tested according to the method of Wei *et*.*al*., [62]. The fungal antagonist was inoculated in TSA medium supplemented with the amino acid glycine (4.4g /L of medium). A strip of sterilized filter paper saturated with a solution containing 0.5% picric acid and sodium carbonate (2%) was placed in the upper half of a Petridish. Each Petridish was then sealed with paraffin and incubated at 30°C for 7 days. A change in color of the filter paper from yellow to brown indicates HCN production. A control set was also incubated with the same experimental condition for each set to determine the color change.

#### Ammonia (NH_3_) production

The production of NH_3_ was qualitatively tested qualitatively according to the method of Wei *et*.*al*.[62]. The antagonist was inoculated in 15 mL of PDA medium in sterilized Petridishes. A strip of sterilized paper saturated with a solution of Hg_2_(Cl_2_)_2_ was placed in the upper half of the Petridish. The Petridishes were then sealed with paraffin and incubated at 28°C for 7 days. A change in colour of the filter paper from white to blackish indicates ammonia production. Two control sets were also incubated with the same experimental condition to determine the color change. One contained sterile PDA medium but was left uninoculated (regarded as the negative control) and in the second control set, 15 mL of sterile NH_4_OH was poured instead of the PDA medium to match the color change (regarded as positive control) in the treated sets as NH_4_OH liberates ammonia gas, which produces about the color change of the filter paper from white to blackish.

### Effect of fungicides on the radial growth of the pathogen

Isolated pathogens were subjected to *in vitro* efficacy on the radial growth of the pathogen with the application of bavistin (2%) and blitox-50 (2%) following the principle of the poison food method. In this assay, 5mm discs of young vigorously growing cultures of pathogens were placed on a 9-cm-diameter sterilized Petridish containing 15 mL of PDA medium mixed with a particular fungicide at the indicated concentration and incubated in a BOD incubator at 28 ± 1°C for 7 days. Control sets were inoculated with 5-mm discs of young vigorously growing cultures of pathogens placed at the center of a plate and incubated under the same conditions but without the fungicide. After 7 days radial growth of the pathogens was recorded for both fungicide treated and control plates. The fungicide activity of each treatment was measured by PIRG. The PIRG was calculated with the following formula [61].

$$PIRG=\frac{\left(A-B\right)}{A\times 100}$$

A = Mean diameter of growth in the control and B = Mean diameter of growth in a given treatment.

### Field trial for *in vivo* study

#### Preparation of spore suspension of fungus for field study

The isolated most potent biocontrol agents were grown in sterilized 1-L conical flasks, each containing 50 g of sterilized substrate (rice husk) and 40 mL of sterile water for 30 days for mass growth. Then, autoclaved distilled water was poured into the conical flasks, and they were shaken with the help of a vortex to prepare the stock spore suspension. The intended spore concentration (10^8^ spores/ mL) was prepared from the stock spore solution with the help of hemocytometer counting.

#### Mini plot design and cultivation techniques

Saplings of *Aloe vera* were planted within 6 ft × 6 ft mini plots. Each mini plot was separated by a 1 ft border. In each plot 25 saplings were planted and tagged, with 5 plants in each row and spacing of 1 ft, between each plant and between each row. A total of 12 plots were arranged in a randomized block design (RBD) and maintained for three consecutive years from 2013 to 2015. For treated plots the saplings were dipped in a spore suspension of *T*. *asperellum*, *T*. *viride* or *T*. *harzianum* (10^8^ spores / mL) for 10 hours before planting. Three plots were kept as untreated controls. Spore suspensions of *T*. *asperellum*, *T*. *viride* and *T*. *harzianum* (each at 10^8^ spores / mL) were sprayed using a knapsack sprayer for the biocontrol of the pathogen in the field at the rate of 2.5 L / plot (three plots for each treatment) 4 times at a 7 day interval for the season. All plants were well maintained with regular irrigation and weeding. In control (untreated) plots, sterile water was sprayed 4 times at a 7 day interval for the season. Continuous monitoring was performed every day during the season in order to identify any symptoms on untreated as well as treated sets. For the study of the PDI per plot, tagged plants were visited regularly, once per week throughout the season for three consecutive years. The PDI was calculated by using the following formula in untreated and treated sets.

$$PDI=\frac{A}{B\times 100}$$

A = Total number of infected plants; B = total number of selected plants.

## Supporting information

S1 Table Occurrence of the leaf spot disease on *Aloe vera* in different regions of North 24 Parganas, West Bengal from 2013–15. (DOCX)

S2 Table Month wise report of the temperature and humidity in study area. (DOCX)

S3 Table Non volatile effects of the fungal biocontrol agents on the radial growth of *Alternaria brassicae*. (DOCX)

S4 Table Volatile effect of the fungal biocontrol agents on radial growth of *Alternaria brassicae*. (DOCX)

S5 Table *In vitro* comparison between applications of fungicides with the most potent biocontrol agents. (DOCX)
