# Supplementary material for: Encountering epidemic effects of leaf spot disease (Alternaria brassicae) on Aloe vera by fungal biocontrol agents in agrifields—An ecofriendly approach
Source: PLoS One. 2018 Mar 26;13(3):e0193720. doi: 10.1371/journal.pone.0193720 (PMC5868775; doi:10.1371/journal.pone.0193720)
Supplement: S1 Table — (DOCX) [file pone.0193720.s001.docx]

**Supporting Information.**

**Supplementary Table**

**S1 Table. Occurrence of the leaf spot disease on *Aloe vera* in different regions of North 24 Parganas, West Bengal from 2013-15**

| **Survey areas** | **Leaf Spot disease of *Aloe vera*** | | |
| --- | --- | --- | --- |
|  | **2013** | **2014** | **2015** |
| Barasat | + | + | + |
| Noihati | + | + | + |
| Basirhat | + | + | + |
| Moishbathan | + | + | + |
| Barrackpore | + | + | + |
| Nilgunj | + | + | + |
| Haroa | + | + | + |
| Basanti | + | + | + |
| Duttapukur | + | + | + |
| Bongaon | + | + | + |
| Habra | + | + | + |
| Kalyani | + | + | + |
| Halishahar | + | + | + |
| Taki | + | + | + |
| Hingalgunj | + | + | + |
| Nahata | + | + | + |
| Gopalnagar | + | + | + |

“+” indicates presence of the leaf spot disease, whereas “-” indicates absence of the disease.
