# Supplementary material for: Encountering epidemic effects of leaf spot disease (Alternaria brassicae) on Aloe vera by fungal biocontrol agents in agrifields—An ecofriendly approach
Source: PLoS One. 2018 Mar 26;13(3):e0193720. doi: 10.1371/journal.pone.0193720 (PMC5868775; doi:10.1371/journal.pone.0193720)
Supplement: S2 Table — (DOCX) [file pone.0193720.s002.docx]

**Supporting Information.**

**Supplementary Table**

**S2 Table. Month wise report of the temperature and humidity in study area**

| **Month** | **Temperature ^ᵒ^C** | | | **Relative Humidity %** | | |
| --- | --- | --- | --- | --- | --- | --- |
|  | **2013** | **2014** | **2015** | **2013** | **2014** | **2015** |
| January | 13-26 | 10-26 | 11-28 | 71-84 | 70-78 | 70-82 |
| February | 18-29 | 16-27 | 16-29 | 75-86 | 72-82 | 70-84 |
| March | 25-34 | 19-33 | 21-34 | 79-88 | 78-90 | 77-89 |
| April | 32-42 | 30-41 | 34-42 | 76-90 | 79-96 | 75-92 |
| May | 30-43 | 33-43 | 29-44 | 75-94 | 80-98 | 79-97 |
| June | 28-38 | 28-39 | 29-40 | 78-96 | 77-100 | 79-100 |
| July | 28-37 | 26-38 | 25-39 | 96-100 | 94-100 | 95-100 |
| August | 30-36 | 25-36 | 27-38 | 89-92 | 86-96 | 87-98 |
| September | 30-35 | 25-35 | 28-37 | 85-92 | 89-95 | 83-97 |
| October | 25-35 | 22-31 | 22-32 | 80-90 | 86-92 | 78-88 |
| November | 20-25 | 20-25 | 20-28 | 70-80 | 70-80 | 70-81 |
| December | 10-20 | 10-20 | 12-27 | 65-70 | 65-70 | 62-73 |
