# Supplementary material for: Encountering epidemic effects of leaf spot disease (Alternaria brassicae) on Aloe vera by fungal biocontrol agents in agrifields—An ecofriendly approach
Source: PLoS One. 2018 Mar 26;13(3):e0193720. doi: 10.1371/journal.pone.0193720 (PMC5868775; doi:10.1371/journal.pone.0193720)
Supplement: S3 Table — (DOCX) [file pone.0193720.s003.docx]

**Supporting Information.**

**Supplementary Table**

**S3 Table. Non volatile effects of the fungal biocontrol agents on the radial growth of *Alternaria brassicae***

| **BCA** | **Average radial growth (cm) of pathogen** | | | **Percentage of Inhibition of Radial growth (PIRG) of antagonistic fungi over *A. brassicae***  **(after 7 days)** | | |
| --- | --- | --- | --- | --- | --- | --- |
|  | R1 | R2 | R3 | R1 | R2 | R3 |
| *T. asperellum* | 2.8 | 2.5 | 2.65 | 68.88 | 72.06 | 70.22 |
| *T. harzianum* | 3.1 | 3.05 | 3.0 | 65.55 | 65.92 | 66.29 |
| *T. viride* | 2.9 | 3.0 | 2.90 | 67.77 | 66.48 | 67.41 |
| *T. longibrachiatum* | 3.2 | 3.1 | 3.15 | 64.44 | 65.36 | 64.60 |
| *Beauveria bassiana* | 3.15 | 3.20 | 3.15 | 65.00 | 64.24 | 64.60 |
| Control | 9.0 | 8.95 | 8.90 |  |  |  |
