# Supplementary material for: Encountering epidemic effects of leaf spot disease (Alternaria brassicae) on Aloe vera by fungal biocontrol agents in agrifields—An ecofriendly approach
Source: PLoS One. 2018 Mar 26;13(3):e0193720. doi: 10.1371/journal.pone.0193720 (PMC5868775; doi:10.1371/journal.pone.0193720)
Supplement: S4 Table — (DOCX) [file pone.0193720.s004.docx]

**Supporting Information.**

**Supplementary Table**

**S4 Table. Volatile effect of the fungal biocontrol agents on radial growth of *Alternaria brassicae***

| **BCA** | **Average radial growth (cm) of pathogen** | | | **Percentage of Inhibition of Radial growth (PIRG) of antagonistic fungi over *A. brassicae***  **(after 7 days)** | | |
| --- | --- | --- | --- | --- | --- | --- |
|  | R1 | R2 | R3 | R1 | R2 | R3 |
| *T. asperellum* | 2.2 | 2.4 | 2.3 | 73.49 | 71.08 | 71.95 |
| *T. harzianum* | 3 | 3.05 | 3.05 | 63.85 | 63.25 | 62.80 |
| *T. viride* | 2.95 | 2.85 | 2.8 | 64.45 | 65.66 | 65.85 |
| *T. longibrachiatum* | 3.5 | 3.35 | 3.2 | 57.83 | 59.63 | 60.97 |
| *Beauveria bassiana* | 5.5 | 5.5 | 5.5 | 33.73 | 33.73 | 32.92 |
| Control | 8.3 | 8.3 | 8.2 |  |  |  |
