# Supplementary material for: Encountering epidemic effects of leaf spot disease (Alternaria brassicae) on Aloe vera by fungal biocontrol agents in agrifields—An ecofriendly approach
Source: PLoS One. 2018 Mar 26;13(3):e0193720. doi: 10.1371/journal.pone.0193720 (PMC5868775; doi:10.1371/journal.pone.0193720)
Supplement: S5 Table — (DOCX) [file pone.0193720.s005.docx]

**Supporting Information.**

**Supplementary Table**

**S5 Table. *in vitro* comparison between applications of fungicides with the most potent biocontrol agent*s***

| **BCA** | **Average radial growth (cm) of pathogen** | | | **Percentage of Inhibition of Radial growth (PIRG) of antagonistic fungi over *A. brassicae***  **(after 7 days)** | | |
| --- | --- | --- | --- | --- | --- | --- |
|  | R1 | R2 | R3 | R1 | R2 | R3 |
| Bavistin  (2% conc.) | 3.6 | 3.7 | 3.5 | 59.55 | 58.88 | 60.89 |
| Blitox-50  (2% conc.) | 2.3 | 2.3 | 2.4 | 74.15 | 74.44 | 73.18 |
| *T. asperellum* | 2.5 | 2.75 | 2.7 | 71.91 | 69.44 | 69.83 |
| *T. harzianum* | 3.1 | 3 | 3.05 | 65.16 | 66.66 | 65.92 |
| *T. viride* | 2.9 | 3 | 2.9 | 67.41 | 66.66 | 67.59 |
| Control (untreated) | 8.9 | 9 | 8.95 |  |  |  |
